# Characterization of full-length long noncoding RNAs and identification of virus-responsive lncRNAs in *Sogatella furcifera*

**DOI:** 10.3389/fmicb.2025.1643735

**Published:** 2025-07-02

**Authors:** Yawen Ban, Ting Cui, Lifei Zhao, Xue Li, Qing Bai, Qingfa Wu

**Affiliations:** ^1^Division of Life Sciences and Medicine, Department of Pharmacy, The First Affiliated Hospital of USTC, University of Science and Technology of China, Hefei, Anhui, China; ^2^Key Laboratory of Anhui Province for Emerging and Reemerging Infectious Diseases, University of Science and Technology of China, Hefei, China

**Keywords:** long non-coding RNA, *Sogatella furcifera*, PacBio SMRT sequencing, developmental regulation, SRBSDV, virus-host interaction, sex-biased expression

## Abstract

Long non-coding RNAs (lncRNAs) are crucial regulators of development and stress responses in eukaryotes, but their roles in non-model insects, particularly rice planthoppers, remain poorly characterized. Here, we present a comprehensive identification and characterization of full-length lncRNAs in the white-backed planthopper (*Sogatella furcifera*), a major rice pest and efficient vector of Southern rice black-streaked dwarf virus (SRBSDV). By integrating PacBio single-molecule real-time (SMRT) sequencing with publicly available Iso-Seq datasets, we reconstructed a high-confidence, full-length transcriptome and identified 1,211 lncRNAs spanning 1,174 loci. These lncRNAs displayed canonical features, including lower GC content, shorter transcript length, and fewer exons. Unlike mRNAs, which exhibited extensive alternative splicing, only 21 splicing events were detected among lncRNAs. Stage-specific lncRNAs were predominantly expressed during embryogenesis (e.g., 93 transcripts between 0 and 72 h post-oviposition) and molting (e.g., 16 in pre-ecdysis), while sex-biased expression patterns emerged after 24 h. Functional enrichment analysis linked male-biased lncRNAs to metabolic pathways and molting-associated lncRNAs to tissue remodeling processes, including Wnt signaling. Upon SRBSDV infection, approximately 50% of differentially expressed lncRNAs responded to both virus exposure (feeding on infected plants) and confirmed viral replication, exhibiting distinct sex-dimorphic regulation. K-means clustering defined four major expression modules: female-biased (Cluster 1), male-biased (Cluster 2), and two virus-suppressed clusters (Clusters 3 and 4), which were significantly enriched for immune-related pathways such as PI3K-Akt signaling and phagosome formation. We further experimentally validated five sex-independent virus-responsive lncRNAs, *sfur_LNC0242*, *sfur_LNC1059*, *sfur_LNC0956*, and *sfur_LNC0346* (upregulated), and *sfur_LNC0483* (downregulated), with predicted involvement in NF-κB signaling, ubiquitin-mediated proteolysis, and metabolic adaptation through AMPK/PPAR pathways. Altogether, this study provides the first full-length lncRNA reference for *S. furcifera* and reveals the dynamic regulation of lncRNAs across development and in response to viral infection. The identification of sex-specific and conserved virus-responsive lncRNAs offers promising molecular targets for disrupting vector competence and advancing RNAi-based pest management strategies.

## 1 Introduction

Long non-coding RNAs (lncRNAs) represent a diverse and underexplored class of the eukaryotic transcriptome, defined as transcripts longer than 200 nucleotides that lack protein-coding potential (Mattick et al., 2023). Despite their non-coding nature, lncRNAs have emerged as critical regulators of gene expression, acting through mechanisms such as chromatin modification, transcriptional regulation, alternative splicing, and post-transcriptional control (Mattick et al., 2023). Functionally, they serve as molecular scaffolds, decoys, and guides, influencing a broad array of biological processes including development, stress responses, and host-pathogen interactions (Dahariya et al., 2019; Chen and Kim, 2024).

Although extensive efforts have been directed toward annotating and characterizing protein-coding genes, the functional landscape of lncRNAs remains comparatively obscure, particularly in non-model organisms. In insects, lncRNA research is still in its early stages. While thousands of lncRNAs have been annotated in species such as *Drosophila melanogaster* (Young et al., 2012; Chen B. et al., 2016; Schor et al., 2018), *Bombyx mori* (Wu et al., 2016; Ruan et al., 2020), *Aedes aegypti* (Azlan et al., 2019) and *Locusta migratoria* (Li et al., 2020), insights into their biological functions, regulatory mechanisms, and roles in development, immunity, and host-pathogen interactions remain limited. Rice planthoppers, including the brown planthopper (*Nilaparvata lugens*), the white-backed planthopper (*Sogatella furcifera*, WBPH), and the small brown planthopper (*Laodelphax striatellus*, SBPH), are among the most destructive insect pests in Asia, causing significant yield losses in rice cultivation. A previous study identified 2,439 lncRNAs in *N. lugens* (Xiao et al., 2015). In our prior short-read–based work, we annotated 1,861 lncRNAs from 1,852 loci in WBPH and 390 lncRNAs from 385 loci in SBPH (Chang et al., 2020). Notably, lncRNA expression in these species is often temporally restricted, with enrichment in embryonic and late nymphal stages, suggesting important roles in development (Chang et al., 2020).

Functional studies of insect lncRNAs have begun to reveal diverse biological roles. In *Drosophila*, the lncRNA *roX* mediates dosage compensation by activating X-linked genes and repressing autosomal targets via interactions with MSL and PRC complexes (Li et al., 2025). LncRNA *CR33938* in *B. mori* shows stage-specific expression during late development (Tse et al., 2022), and *Bmdx-AS1* regulates male genitalia development (Wang et al., 2022). In agricultural pests such as *Plutella xylostella* and *Helicoverpa armigera*, lncRNAs modulate detoxification gene expression (Wang et al., 2018). LncRNAs also respond to environmental stress; for example, *Hsrω* in *Drosophila* promotes intestinal stem cell differentiation under heat stress (Guo et al., 2024). Furthermore, emerging evidence highlights the involvement of lncRNAs in host-virus interactions. These RNAs can modulate antiviral responses by directly interacting with immune signaling components (e.g., NF-κB, IRFs), functioning as competing endogenous RNAs (ceRNAs) to sequester antiviral microRNAs, or associating with viral/host proteins to regulate gene expression. Chromatin remodeling by lncRNAs can also activate or suppress antiviral genes. For instance, *Bombyx mori* nucleopolyhedrovirus (BmNPV) represses *Lnc_209997*, promoting *miR-275-5p* expression and facilitating viral replication (Lin et al., 2022). Other differentially expressed lncRNAs in *B. mori* modulate host responses to BmNPV by interacting with target genes and miRNAs. *LINC5438* suppresses apoptosis in silkworm cells (Chen et al., 2023), while the *A. aegypti* lncRNA *Zinc22*, enriched in ovaries, influences both reproduction and antiviral resistance (Belavilas-Trovas et al., 2023), suggesting a metabolic trade-off. In *Drosophila*, *VINR* activates non-canonical innate immunity upon detecting the viral suppressor of RNAi (VSR) from *Drosophila C virus* (DCV) (Zhang L. et al., 2020), and *CR46018* enhances the Toll pathway via interaction with Dif and Dorsal, independent of microbial infection (Zhou et al., 2021).

In this study, we generated a high-quality, full-length transcriptome of WBPH by integrating PacBio SMRT sequencing. This comprehensive approach enabled the accurate annotation of lncRNAs, including their full-length structures, alternative splicing isoforms, and numerous novel transcripts that were not detectable using short-read sequencing methods. Leveraging these improved annotations, we analyzed lncRNA expression dynamics across developmental stages and identified stage-specific and functionally relevant lncRNAs. Notably, we uncovered both sex-biased and virus-responsive transcriptional modules associated with SRBSDV infection and further characterized a subset of sex-independent, virus-responsive lncRNAs in *WBPH*. Collectively, this study provides the first full-length lncRNA reference for WBPH and establishes a foundational resource for future functional investigations of lncRNAs in planthoppers.

## 2 Materials and methods

### 2.1 Identifications of lncRNAs

To comprehensively identify full-length splice variants of long non-coding RNAs (lncRNAs) in *S. furcifera*, two libraries from PacBio Iso-Seq platform for full-length transcriptome sequencing through Biomarker Technologies Corporation (Beijing, China), were applied for the comprehensive lncRNAs characterization. Raw reads were processed using Trimmomatic (v0.39) (Bolger et al., 2014) to remove adapter sequences and low-quality bases. Cleaned reads were assembled into transcripts using StringTie (v2.1.4) (Pertea et al., 2015) with a reference genome-guided approach and assembled transcripts were compared against the NCBI non-redundant (nr) protein database using BlastX (*E*-value < 1e−5) to filter out potential protein-coding sequences. Transcripts with open reading frames (ORFs) longer than 100 amino acids were excluded using TransDecoder (v5.5.0). Only transcripts longer than 200 nucleotides (nt) were retained for further analysis. The gffcompare tool was used to classify transcripts, and only those with class codes “u,” “x,” “i,” “j,” and “o” (indicating novel lncRNAs) were selected. The Coding-Non-Coding Index (CNCI) (Luo et al., 2014) and Coding Potential Calculator (CPC) (Kang et al., 2017) were employed to further exclude transcripts with coding potential. Transcripts containing known protein domains (Pfam database, *E*-value < 0.001) were discarded. Known housekeeping RNAs (e.g., rRNAs, tRNAs) were filtered out using the Rfam database. Only transcripts with FPKM > 1 in at least one sample were retained as high confidence lncRNAs.

### 2.2 Developmental stage-specific expression analysis

The 45 publicly available RNA-seq libraries of WBPH from NCBI were applied to comprehensively analyze the features of these 1,211 lncRNAs, including 33 libraries covering various developmental stages and 12 libraries representing different tissues. Due to substantial heterogeneity among samples from different sources, we performed independent analyses for each BioProject without batch correction or merging datasets. This approach ensured that variations introduced by different experimental conditions or sequencing platforms did not confound our results. Developmental stage-specific lncRNAs were identified through differential expression analysis (DESeq2, FDR < 0.05, |log_2_FC| > 1) (Liu et al., 2021). First, we calculated the Tau (tissue-specific gene expression) (Lüleci and Yılmaz, 2022) values for different tissues and developmental stages of the brown planthopper separately. The formula used is as follows:


$$\tau =\frac{\sum_{i=1}^{N}(1-\mathrm{Xi})}{N-1}$$

Here, “*N*” represents the number of developmental stage groups, and “*Xi*” denotes the expression level of a gene in a specific tissue or developmental stage sample (normalized by the maximal component value). For samples with replicated data, the average expression for each stage was calculated first before computing the Tau value. The Tau value ranges between 0 and 1, where a lower Tau value (closer to 0) indicates higher constitutive expression, and a higher Tau value (closer to 1) indicates higher stage-specific expression. Finally, we filtered genes based on the criteria Tau > 0.8 and log_10_ (max exp. (TPM) > 0). Subsequently, we performed differential expression analysis on the data from different developmental stages of *S. furcifera* to identify specifically highly expressed genes. For each specific developmental stage, the expression data were treated as the experimental group, while the remaining samples served as the control group. Differential expression analysis was conducted using the DESeq2 software, and genes were filtered based on the criteria FDR < 0.05 and |log_2_FC| > 1. For the specifically highly expressed genes obtained from both methods, duplicate genes were removed to yield the final set of developmental stage-specific highly expressed genes.

### 2.3 LncRNA-mRNA regulatory networks

Potential cis-acting lncRNAs were predicted based on genomic proximity (< 100 kb upstream/downstream of neighboring genes). Co-expression networks were constructed using WGCNA to identify trans-regulatory interactions (Langfelder and Horvath, 2008; Tian et al., 2020). Target genes of lncRNAs were subjected to GO and KEGG enrichment analyses. Module analysis was performed to identify key functional clusters. First, based on the genomic location information of lncRNAs, we used the bedtools tool to extract mRNAs within 100 kb upstream and downstream of each lncRNA as the gene set for cis-regulation by lncRNAs. Subsequently, extract the expression profiles of lncRNAs and mRNAs, and use the Spearman method of the rcorr function in the Hmisc package to calculate the expression correlation between each lncRNA-mRNA pair, as well as the reliability *p*-value. Screen for lncRNA-mRNA relationship pairs with cor > 0.95 and FDR < 0.05 as the gene set for trans-regulation by lncRNAs. For the target mRNA gene sets regulated by developmental stage-specific lncRNAs in rice planthoppers, we performed functional enrichment analysis using the clusterProfiler package.

### 2.4 Screening and functional validation of virus-responsive lncRNAs

Healthy individuals of *S. furcifera* were collected from southern Anhui Province, China, and subsequently maintained in the laboratory for several generations under controlled conditions. SRBSDV-infected rice plants were initially collected from Guangdong Province and subsequently propagated under greenhouse conditions through transmission by *S. furcifera*. A comprehensive transcriptomic analysis were performed for 21 *S. furcifera* individual samples included three experimental groups: (i) virus-free planthoppers feeding on healthy rice plants, comprising virus-free males (VFM) and virus-free females (VFF), which served as controls; (ii) virus-infected planthoppers feeding on SRBSDV-infected rice plants with confirmed viral presence as detected by RT-PCR, including virus-infected males (VIM) and females (VIF); and (iii) virus-exposed planthoppers feeding on SRBSDV-infected plants but lacking detectable virus based on RT-PCR, including virus-exposed males (VEM) and females (VEF) (Supplementary Table S6). Virus-infected vs. control *S. furcifera* were compared to identify differentially expressed lncRNAs (FDR < 0.05, |log_2_FC| > 1). Target genes of virus-responsive lncRNAs were analyzed for enrichment in antiviral pathways. Key lncRNAs were selected for co-expression network analysis to predict their regulatory roles. The RT-qPCR was used to confirm differential expressions of candidate lncRNAs. To quantify lncRNAs expression, total RNA was extracted from 6 experimental groups, with each group consisting of 10 planthoppers. RT-qPCR assays were performed using the ToloScript All-in-one RT EasyMix (TOLOBIO, 22107). The actin transcript of *S. furcifera* served as the internal reference, and the relative levels of lncRNA expression were calculated using the 2^−ΔΔCT^ method. Primer sequence q-Actin-F: GCCGTCTTTCTTGGGTATGG, q-Actin-R: AGGGCGGTGATCTCCTTCTG.

### 2.5 Functional and pathway analysis

All of the differentially expressed genes (ranked by *p*-value) (Supplementary Table S6) were subjected to Gene Ontology (GO) (The Gene Ontology Consortium, 2019) and Kyoto Encyclopedia of Genes and Genomes (KEGG) (Kanehisa et al., 2017) pathway enrichment analyses to elucidate the biological functional characteristics of the clusters (Chen et al., 2017).

### 2.6 Statistical analysis

All the quantitative data presented in the text and figures were analyzed with two-tailed *t*-tests in GraphPad Prism 9 (GraphPad Software, San Diego, CA, United States). The other statistical analyses were conducted in R (v4.1.0), and visualization was performed using ggplot2 and Cytoscape (v3.8.2).

## 3 Results

### 3.1 Characterization of lncRNAs using PacBio SMRT sequencing

**Figure 1:**
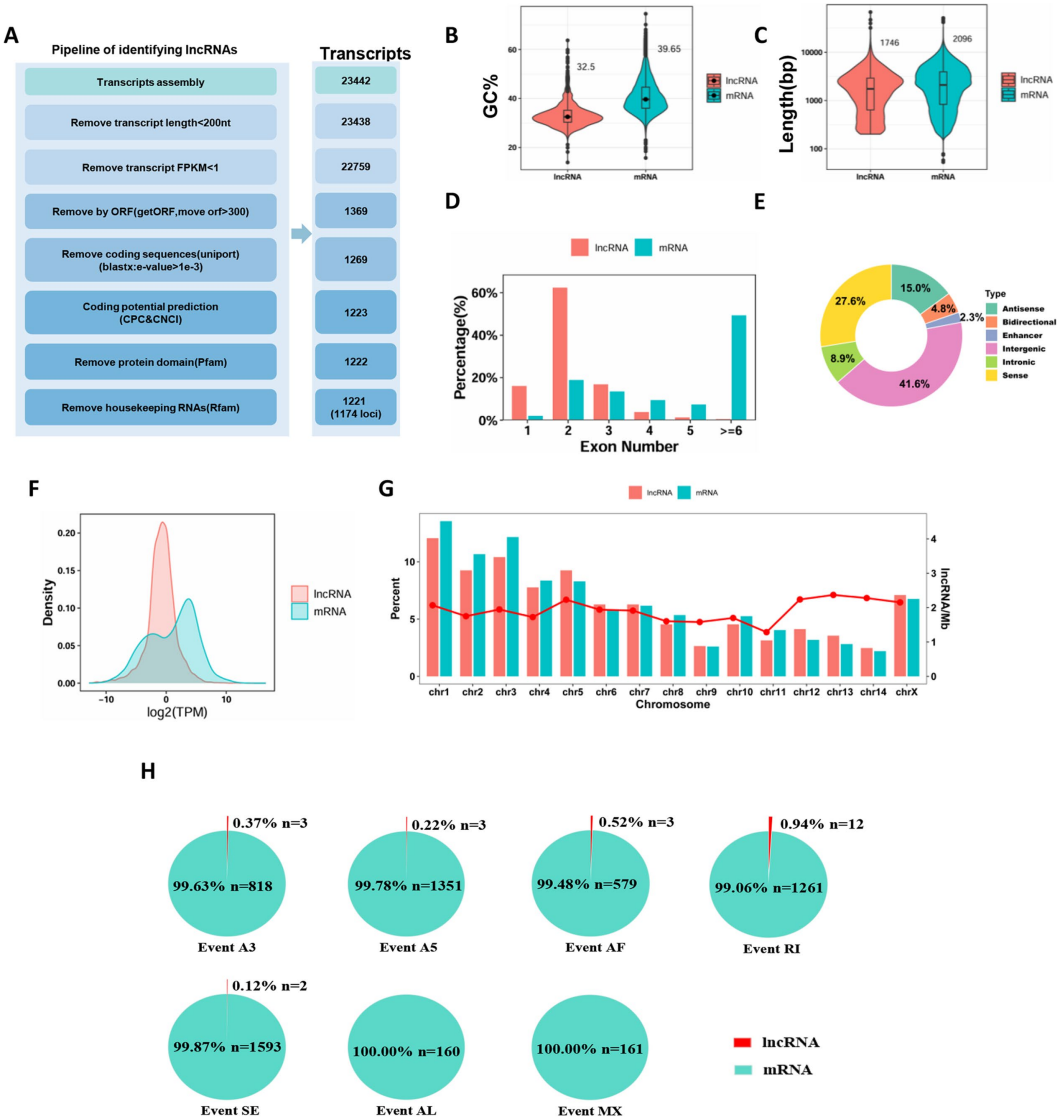
Characteristic comparison among full-length transcriptome-identified lncRNAs and all mRNAs in *Sogatella furcifera*. **(A)** Schematic diagram of the bioinformatics pipeline for lncRNA prediction. **(B–G)** The GC content, length, exon numbers, classification, chromosomal distribution and expression profiles of mRNAs and lncRNAs in *S. furcifera*. **(H)** The distribution of all alternative splicing event types in *S. furcifera*.

To comprehensively identify full-length splice variants of lncRNAs in the *S. furcifera*, we employed the PacBio Iso-Seq platform for full-length transcriptome sequencing on total RNA extracted from individuals across all developmental stages (Supplementary Table S1). In total, we obtained 192,878 Iso-Seq transcript sequences, with an average length of 4,665 bp. To enhance the coverage and accuracy of lncRNA identification, we also incorporated publicly available Iso-Seq data (NCBI SRA: PRJNA561275) (Chen et al., 2020) into our integrated analysis (Supplementary Table S1). This project yielded a total of 251,109 Iso-Seq transcript sequences, with an average length of 2,682 bp. Combined analysis of these full-length transcriptome datasets identified 1,211 lncRNA transcripts originating from 1,174 gene loci (Figure 1A; Supplementary Table S2), representing the most comprehensive set of full-length transcript isoforms to date and offering a significant improvement over previous annotations based on short-read RNA-seq data.

The identified lncRNAs displayed distinct characteristics compared to messenger RNAs (mRNAs). Notably, the GC content of lncRNAs was significantly lower, with a mean GC content of 32.5%, compared to 39.65% in mRNAs (Figure 1B). In terms of transcript structure, lncRNAs exhibited a shorter average transcript length (1,746 nucleotides) than mRNAs (2,096 nucleotides), and generally contained fewer exons (Figure 1C). The exon number distribution of lncRNAs followed a unimodal pattern, with the majority (754 transcripts, 62.3%) containing two exons. In contrast, mRNAs showed a bimodal exon distribution, including a minor peak at two exons, but with nearly half (19,988 transcripts, 49.2%) containing six or more exons (Figure 1D). To further characterize the genomic origins of these lncRNAs, we classified them based on their positional relationships to protein-coding genes. The majority were intergenic lncRNAs (525, 41.6%), followed by sense (348, 27.6%), antisense (189, 15.0%), intronic (112, 8.9%), bidirectional (60, 4.8%), and enhancer-associated lncRNAs (29, 2.3%) (Figure 1E; Supplementary Table S2). We also analyzed the expression profiles of lncRNAs and mRNAs using normalized expression values derived from the full-length transcriptome data. Overall, lncRNAs exhibited significantly lower expression levels than mRNAs. The expression distribution of lncRNAs followed a unimodal pattern, while mRNAs displayed a bimodal distribution: one peak overlapping the lower expression levels of lncRNAs, and a second peak representing transcripts with much higher expression levels (Figure 1F). The expression levels of lncRNAs show a unimodal distribution due to their functional and transcriptional regulatory characteristics, while those of mRNAs exhibit a bimodal distribution because of the differences in gene functions and the complexity of post-transcriptional regulation. Although the absolute number of lncRNAs varied significantly across chromosomes, the density of lncRNAs (i.e., number per megabase) was relatively uniform (Figure 1G).

### 3.2 Detection of alternative splicing events of WBPH lncRNA and mRNA

Alternative splicing (AS) is a fundamental regulatory mechanism in eukaryotic gene expression that significantly expands transcriptomic and proteomic diversity, primarily comprising seven major types of AS events: alternative 3′ splice sites (A3), alternative 5′ splice sites (A5), alternative first exon (AF), alternative last exon (AL), mutually exclusive exons (MX), retained introns (RI), and skipped exons (SE) (Zhang H. et al., 2020). To comprehensively investigate the role of AS in *Sogatella furcifera*, we leveraged a comprehensive collection of RNA-seq datasets derived from *Sogatella furcifera* (Supplementary Table S1). These datasets demonstrated high mapping quality and genomic alignment rate of 91.19% (range: 82.90–94.34%) (Supplementary Table S3), based on alignment to our previously published high-quality, chromosome-level genome assembly (Cui et al., 2023). We systematically analyzed alternative splicing events in both lncRNAs and mRNAs. A total of 4,676 AS events were identified, with the most frequent types being skipped exons (SE; 1,595 events), alternative 5′ splice sites (A5; 1,354 events), and retained introns (RI; 1,261 events), followed by alternative 3′ splice sites (A3; 821), alternative first exons (AF; 581), mutually exclusive exons (MX; 161), and alternative last exons (AL; 160) (Figure 1H). Notably, the vast majority of these AS events occurred in mRNAs, comprising 99.6% of A3 events, 99.8% of A5, 99.5% of AF, and 100% of AL and MX events, as well as 99.1% of RI and 99.9% of SE events. In contrast, AS events in lncRNAs were rare, with only 21 detected across all types. These included 3 A3 events, 3 A5, 3 AF, 12 RI, and 2 SE events (Figure 1H). These findings suggest that while alternative splicing plays a major role in the diversification of protein-coding transcripts, it has a more limited role in the structural variability of lncRNAs in *S. furcifera*.

### 3.3 Developmental stage-specific expression patterns of lncRNAs in WBPH

**Figure 2:**
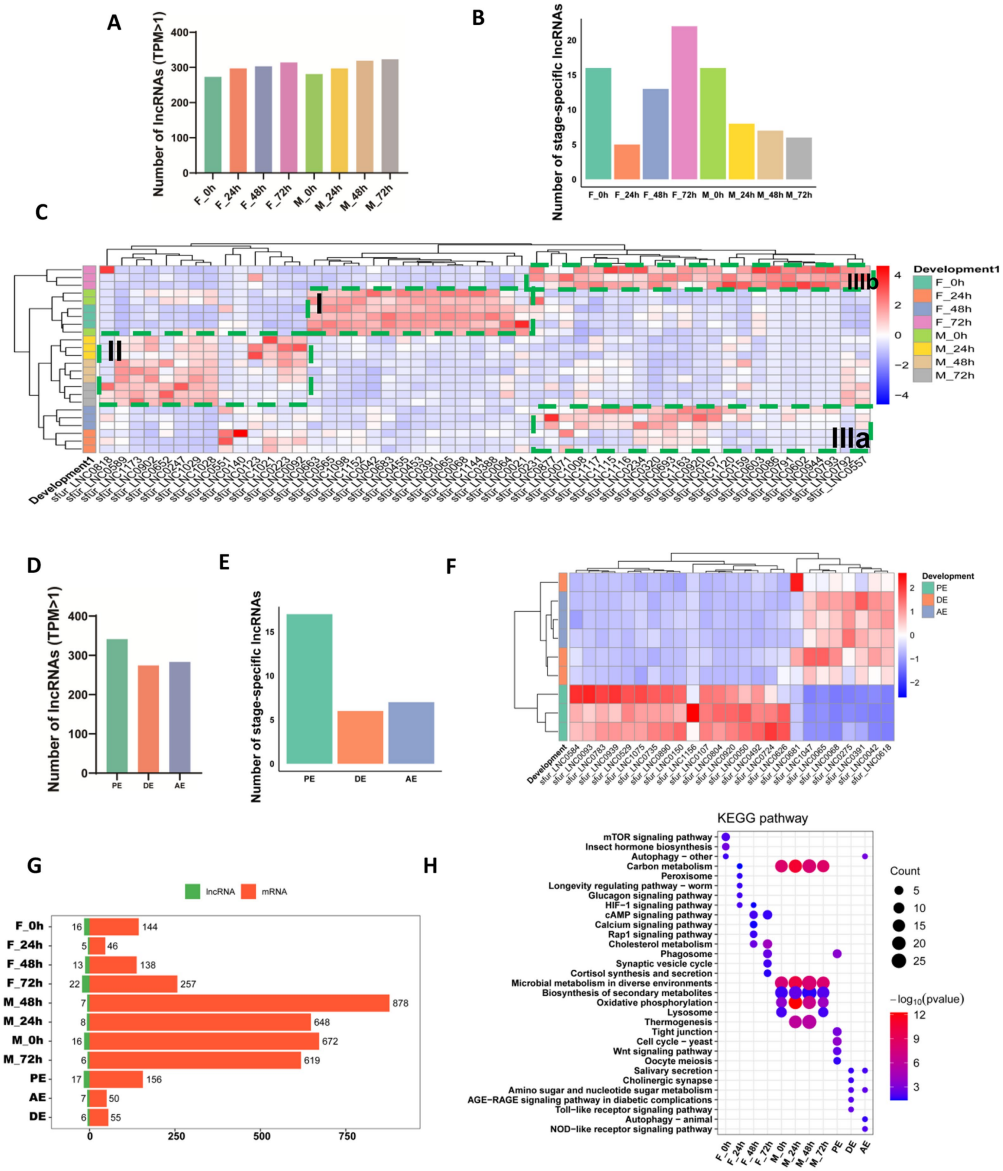
Developmental stage-specific expression patterns of lncRNAs in *Sogatella furcifera*. **(A)** Distribution of the number of highly expressed lncRNAs of early embryogenesis (TPM > 1) in *S. furcifera*. **(B)** Distribution of stage-specific expressed lncRNAs among early embryogenesis stages (Tau > 0.8 and log_2_FC > 1 with *p* < 0.05) in *S. furcifera*. **(C)** Heatmap of expression profiles of stage-specific expressed lncRNAs among early embryogenesis stages from **(B)** in *S. furcifera*. **(D)** Distribution of highly expressed lncRNAs among ecdysis stages (TPM > 1) in *S. furcifera*. **(E)** Distribution of stage-specific expressed lncRNAs among ecdysis stages (Tau > 0.8 and log_2_FC > 1 with *p* < 0.05) in *S. furcifera*. **(F)** Heatmap of expression profiles of stage-specific expressed lncRNAs among ecdysis stages from **(E)** in *S. furcifera*. **(G)** Stage-specific expressed lncRNAs in *S. furcifera* and the distribution of cis/trans-regulated mRNA counts. **(H)** Highly expressed lncRNAs specific to each developmental stage of *S. furcifera* and KEGG pathway enrichment analysis of their potentially regulated mRNAs.

To assess whether lncRNAs play important role in development of WBPH, we examined the expression profiles in both male and female embryos during early embryogenesis stages, specifically at 0 h (F_0h, M_0h), 24 h (F_24h, M_24h), 48 h (F_48h, M_48h), and 72 h (F_72h, M_72h). To account for differences in sequencing depth across libraries, transcript expression levels were normalized using the Transcript per Kilobase per Million mapped reads (TPM) for both mRNAs and lncRNAs. To ensure the reliability of downstream analyses, we focused on lncRNAs with robust expression, defined as transcripts exhibiting ≥1 TPM in at least one developmental stage. A total of 412 lncRNAs met this criterion and were retained for further analysis during early embryogenesis. Specifically, in females, 273 lncRNAs were expressed at 0 h, increasing to 297 at 24 h, 306 at 48 h, and 314 at 72 h. In males, 281 lncRNAs were detected at 0 h, followed by 297 at 24 h, 319 at 48 h, and 323 at 72 h (Figure 2A).

To investigate stage-specific expression patterns of lncRNAs during early embryogenesis in *S. furcifera*, we first applied the Tau index, a tissue-specific gene expression score that quantifies the degree of expression specificity across developmental stages. Tau values range from 0 (ubiquitous expression) to 1 (highly stage-specific expression). lncRNAs with Tau > 0.8 and a maximum expression level of log₁₀(TPMₘₐₓ) > 0 were defined as developmentally stage-specific. Based on this criterion alone, 13 lncRNAs were identified, with most stages exhibiting 0–2 specific lncRNAs; notably, the female 72-h stage (F-72 h) accounted for six of these (Supplementary Figures S1A–C). To broaden the identification of stage-specific lncRNAs, we also performed differential expression analysis using DESeq2, with thresholds of |log₂FC| > 1 and *p*-value < 0.05 (Supplementary Figures S1D,E). We then integrated the results from both the Tau-based analysis and the differential expression approach, generating a comprehensive list of 93 stage-specific lncRNAs across early embryogenesis. Specifically, 16 lncRNAs were stage-specific in females and 16 in males at 0 h, 5 in females and 8 in males at 24 h, 13 in females and 7 in males at 48 h, and 22 in females and 6 in males at 72 h (Figure 2B; Supplementary Table S4). To visualize expression dynamics, hierarchical clustering of these lncRNAs across developmental time points revealed distinct stage-and sex-specific expression patterns (Figure 2C). Notably, a group of lncRNAs exhibited conserved stage specificity at the 0-h time point in both sexes (F_0h and M_0h) (Figure 2C, Box I). Among them, 15 lncRNAs, including *sfur_LNC0565*, *sfur_LNC0068*, and *sfur_LNC0388,* formed a distinct cluster marked by high expression at 0 h followed by sharp downregulation at later stages. In contrast, stage-specific lncRNAs expressed at 24, 48, and 72 h segregated by sex in the clustering analysis, suggesting divergent regulatory programs in male and female embryos (Figure 2C, Box II for males; Boxes IIIa and IIIb for females). These findings underscore the temporally dynamic and sex-specific regulation of lncRNAs during embryogenesis and suggest potential roles for these transcripts in developmental timing and reproductive differentiation in *S. furcifera*.

Extending this approach, we next examined stage-specific lncRNA expression during key molting phases—pre-ecdysis (PE), ecdysis (DE), and post-ecdysis (AE) (Supplementary Figures S1F–J). In total, 380 robustly expressed lncRNAs were detected across these molting stages, with 283, 274, and 341 lncRNAs observed in PE, DE, and AE, respectively (Figure 2D). Among these, 16 lncRNAs were identified as stage-specific in PE based on either the Tau index or differential expression analysis, whereas only 7 and 6 stage-specific lncRNAs were identified in AE and DE, respectively (Figure 2E). Hierarchical clustering revealed that lncRNA expression profiles during DE and AE were more similar to each other, but distinctly different from those observed in PE (Figure 2F), suggesting that lncRNAs may contribute to specific molecular programs associated with the initiation of the molting process.

LncRNAs can regulate mRNA expression through both cis-and trans-acting mechanisms. Cis-acting lncRNAs influence the expression of neighboring genes located within 100 kb upstream or downstream by interacting with chromatin or recruiting transcriptional regulators. In contrast, trans-acting lncRNAs regulate distant genes by forming RNA–RNA or RNA–protein interactions, often inferred through strong expression correlations (Spearman correlation coefficient > 0.95) (Yan et al., 2017; Gil and Ulitsky, 2020). Following these principles, we identified putative cis and trans mRNA targets of the stage-specific lncRNAs during early embryogenesis and molting phases. Interestingly, the total number of predicted mRNA targets during male embryogenesis was substantially higher than in females, despite more stage-specific lncRNAs being identified in female embryos (Figure 2G). KEGG pathway enrichment analysis revealed that the mRNAs potentially regulated by lncRNAs in males were consistently associated with key metabolic processes, including carbon metabolism, microbial metabolism in diverse environments, biosynthesis of secondary metabolites, and oxidative phosphorylation, from 0 to 72 h of embryogenesis (Figure 2H). In contrast, the predicted mRNA targets of lncRNAs in females exhibited more dynamic pathway associations across developmental time points, with enriched KEGG terms differing markedly between 0, 24, 48, and 72 h. Furthermore, we observed significantly higher levels of gene expression modulation during the pre-ecdysis phase compared to the during-and post-ecdysis stages (Figure 2G). KEGG enrichment analysis revealed that the Tight junction, Cell cycle, and Wnt signaling pathways were most active prior to ecdysis, suggesting that lncRNAs may play a critical role in epithelial remodeling and reinforcing cell–cell adhesion to maintain tissue integrity during molting (Figure 2H). In contrast, pathways related to salivary secretion were enriched during and after ecdysis, indicating that lncRNA-regulated mRNAs may facilitate tissue remodeling through secreted enzymes such as proteases, which could aid in the breakdown of old cuticle components (Figure 2H). Notably, autophagy-related pathways were highly enriched across apolysis-ecdysis (AE) stages, implying lncRNA-mediated regulation of resource recycling, stress response, and developmental plasticity following molting (Figure 2H). Together, these findings underscore the potential functional importance of lncRNAs in orchestrating the complex molecular events underlying WBPH early embryogenesis and molting phases.

### 3.4 Global expression profiling revealed sex-biased and virus-responsive transcriptional modules

The white-backed planthopper serves as a highly efficient insect vector of SRBSDV, transmitting the virus in a persistent-propagative manner. To investigate whether lncRNAs are involved in the host response to SRBSDV infection, we performed a comprehensive transcriptomic analysis of 21 *S. furcifera* individual samples. These samples included three experimental groups: (i) virus-free planthoppers feeding on healthy rice plants, comprising virus-free males (VFM) and virus-free females (VFF), which served as controls; (ii) virus-infected planthoppers feeding on SRBSDV-infected rice plants with confirmed viral presence as detected by RT-PCR, including virus-infected males (VIM) and females (VIF); and (iii) virus-exposed planthoppers feeding on SRBSDV-infected plants but lacking detectable virus based on RT-PCR, including virus-exposed males (VEM) and females (VEF) (Supplementary Table S5).

**Figure 3:**
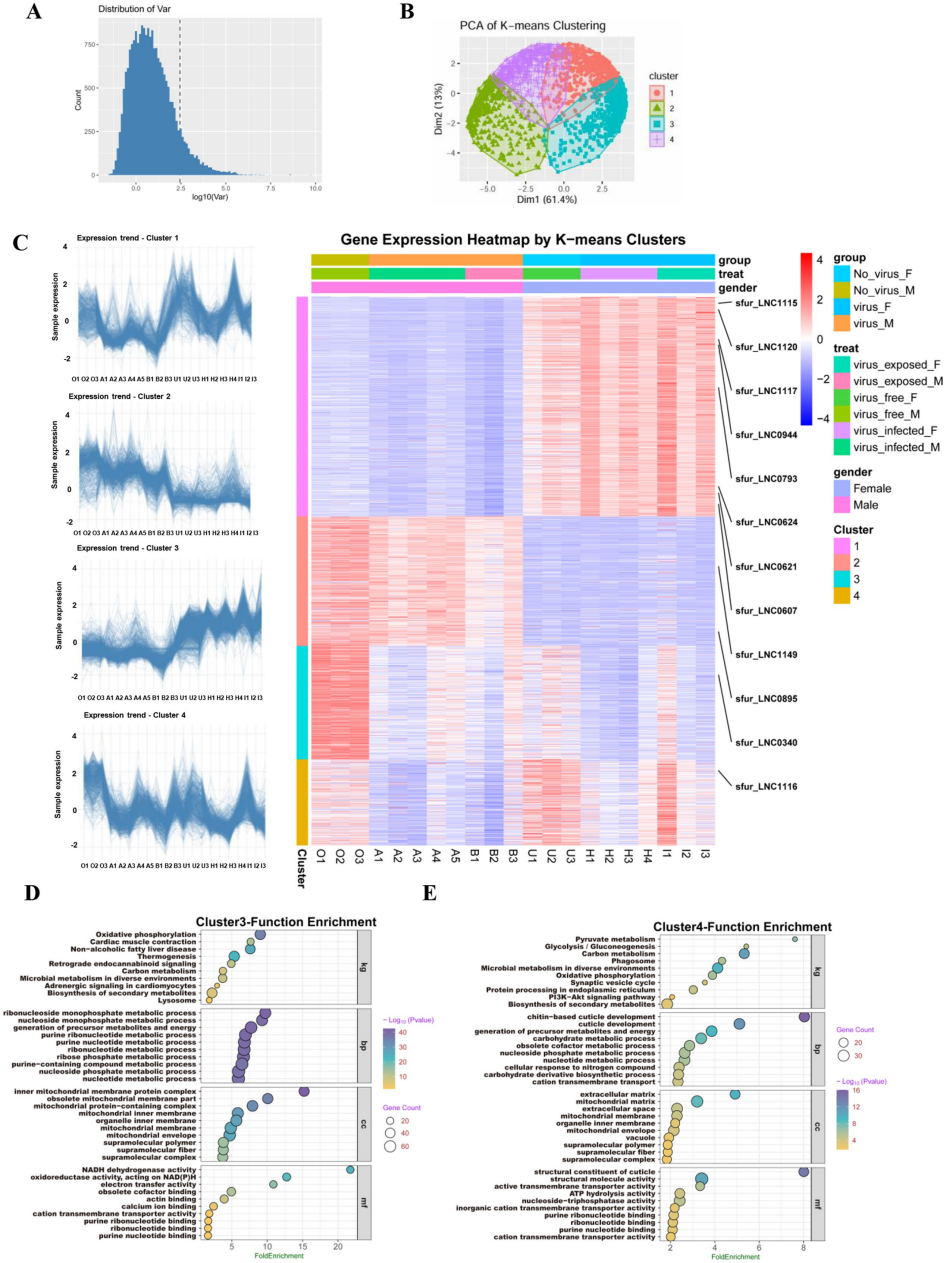
Expression profiles of lncRNAs by k-mean cluster analysis in *S. furcifera* upon the SRBSDV infection. **(A)** The expression variation analysis for screening the lncRNAs for k-means analysis. **(B)** The PCA plot for k-means clustering, with the first principal component (PC1) accounting for 61.4% of the total variance, indicating strong underlying structure in the data and reinforcing the distinct transcriptional programs activated under different experimental conditions. **(C)** Expression trend graphs of lncRNAs in each cluster, with the sample names listed on the x-axis (left) and the heatmap of gene expression in each cluster were revealed in the right graph. **(D,E)** The gene enrichment analysis using KEGG for Cluster 3 and Cluster 4.

To better understand the global transcriptional variation across all experimental conditions, we calculated the standard deviation of expression levels for each mRNA and lncRNA across the 21 *S. furcifera* samples. We then focused on the top 10% mRNAs and lncRNAs (2,119) showing the highest variability, reasoning that these transcripts are likely to be the most responsive to biological perturbations such as virus exposure, infection, and sex-specific regulation (Figure 3A). Using this subset of highly variable genes, we performed K-means clustering and principal component analysis (PCA) to identify patterns of coordinated expression. K-means clustering revealed four distinct gene expression modules, consisting of 848 (840 mRNAs and 8 lncRNAs), 501 (499 mRNAs and 2 lncRNAs), 438 (437 mRNAs and 1 lncRNA), and 332 (331 mRNAs and 1 lncRNA) transcripts, respectively. PCA further supported this classification, with the first principal component (PC1) accounting for 61.4% of the total variance, indicating strong underlying structure in the data and reinforcing the distinct transcriptional programs activated under different experimental conditions (Figure 3B).

Hierarchical clustering and heatmap visualization further revealed that these four gene modules exhibit striking sex-specific expression profiles (Figure 3C). Cluster 1 showed female-biased high expression levels across conditions, including all eight of its lncRNAs (e.g., *sfur_LNC0607*, *sfur_LNC0621*, *sfur_LNC1115*, and *sfur_LNC1120*), regardless of viral infection status. In contrast, Cluster 2 displayed male-biased higher expression regardless of viral infection status, including two lncRNAs (*sfur_LNC0895* and *sfur_LNC1149*). Cluster 3 (containing *sfur_LNC0340*) was characterized by high expression in virus-free males and moderate expression in virus-free females, but markedly reduced expression upon feeding on SRBSDV-infected plants. Similarly, Cluster 4 (containing *sfur_LNC1116*) showed high expression in virus-free females and moderate expression in virus-free males, but significant downregulation following viral infection. Functional enrichment analysis using KEGG pathways revealed that Clusters 3 and 4, which were responsive to viral infection, were significantly enriched in membrane-associated and immune-relevant pathways, including PI3K-Akt signaling, phagosome formation, proteostasis, and secondary metabolite biosynthesis, pathways known to be involved in virus-host interactions and viral replication dynamics (Figures 3D,E). By contrast, Clusters 1 and 2, which displayed clear sex-biased but virus-independent expression patterns, showed no significant enrichment for pathways associated with viral processes (Supplementary Figure S2).

### 3.5 Transcriptomic profiling reveals differential and overlapping lncRNA responses to SRBSDV exposure and infection

The analyses above revealed that lncRNA expression is regulated by a complex interplay of independent factors, including host physiological responses to viral replication, feeding on SRBSDV-infected plants, and biological sex (Supplementary Tables S6, S7). To dissect the individual and combined effects of these factors, we identified differentially expressed genes (DEGs) by performing pairwise comparisons within each sex: virus-free females (VFF) vs. virus-exposed females (VEF) vs. virus-infected females (VIF), and virus-free males (VFM) vs. virus-exposed males (VEM) vs. virus-infected males (VIM), respectively.

**Figure 4:**
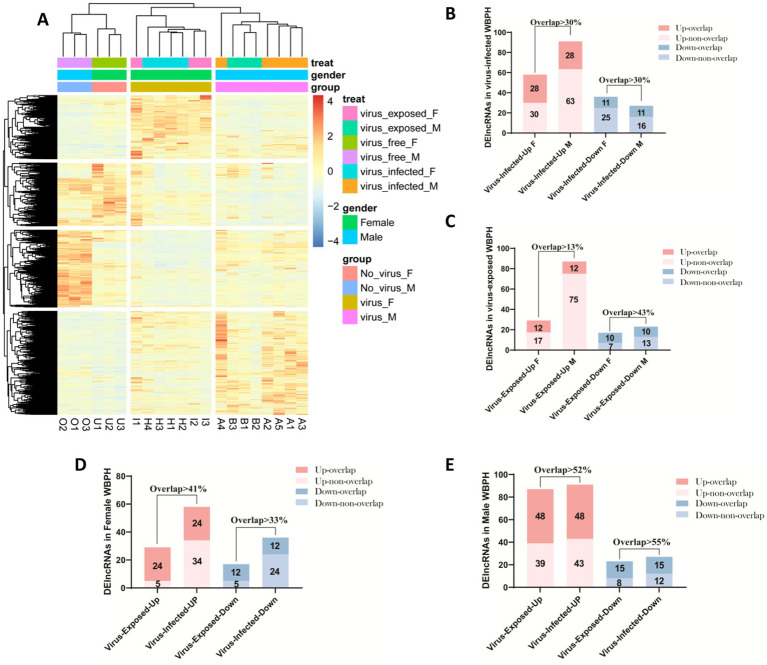
Expression profiles of lncRNAs in *Sogatella furcifera* upon the SRBSDV infection. **(A)** Distribution of the number of differentially expressed lncRNAs among different groups in *S. furcifera*. **(B)** Heatmap of expression profiles of differentially expressed lncRNAs in *S. furcifera* upon SRBSDV infection. **(C–E)** The Histogram plots for the overlapped lncRNAs expression among different groups in *S. furcifera.* Totally 21 samples classified into three experimental groups were included: (i) virus-free planthoppers feeding on healthy rice plants, comprising virus-free males (VFM) and virus-free females (VFF), which served as controls; (ii) virus-infected planthoppers feeding on SRBSDV-infected rice plants with confirmed viral presence as detected by RT-PCR, including virus-infected males (VIM) and females (VIF); and (iii) virus-exposed planthoppers feeding on SRBSDV-infected plants but lacking detectable virus based on RT-PCR, including virus-exposed males (VEM) and females (VEF).

Compared to the virus-free control group, we identified distinct sets of differentially expressed lncRNAs in virus-exposed and virus-infected *WBPH* samples. Specifically, 87 lncRNAs were upregulated and 23 downregulated in virus-exposed males (VEM), while 29 were upregulated and 17 downregulated in virus-exposed females (VEF). In virus-infected individuals, 91 upregulated and 27 downregulated lncRNAs were detected in males (VIM), and 58 upregulated and 36 downregulated in females (VIF) (Supplementary Table S6). Notably, the number of upregulated lncRNAs was significantly higher in virus-exposed and virus-infected males than in their female counterparts, indicating a sex-biased regulatory response to SRBSDV exposure and infection. To further investigate expression patterns, we performed hierarchical clustering of the 21 samples based on the expression profiles of all differentially expressed lncRNAs. Virus-free controls (VFM and VFF) clustered closely together but formed distinct male and female subclusters, while clearly separating from virus-exposed and virus-infected groups. This suggests that feeding on SRBSDV-infected rice plants substantially alters lncRNA expression, with effects that surpass the inherent sex-based expression differences observed under normal conditions. Among virus-exposed and virus-infected samples, we observed distinct clustering by sex, highlighting persistent sex-biased lncRNA regulation even under identical viral treatment. However, the virus-exposed and virus-infected groups did not form entirely separate clusters, implying that both the physiological response to feeding on virus-infected plants and the replication of SRBSDV contribute to lncRNA expression changes (Figure 4A).

To further investigate the regulatory mechanisms underlying lncRNA expression in response to SRBSDV, we examined the overlap of differentially expressed lncRNAs across treatment groups. Identifying commonly regulated lncRNAs may indicate shared upstream regulatory pathways or biological processes. In the virus-infected groups (VIM and VIF), we identified 28 commonly upregulated and 11 commonly downregulated lncRNAs, suggesting these transcripts may be regulated by a combination of host physiological responses to viral replication and the act of feeding on SRBSDV-infected plants, irrespective of sex (Figure 4B). Similarly, in the virus-exposed groups (VEM and VEF), we detected 12 overlapping upregulated and 10 overlapping downregulated lncRNAs (Figure 4C). These lncRNAs are likely responsive primarily to the physiological effects of feeding on virus-infected plants, in the absence of detectable viral replication. Further comparison revealed 24 commonly upregulated and 12 commonly downregulated lncRNAs shared between female groups (VIF and VEF) (Figure 4D), and 48 upregulated and 15 downregulated lncRNAs shared between male groups (VIM and VEM) (Figure 4E). These findings reinforce a sex-biased transcriptional response to SRBSDV-infected feeding plants.

### 3.6 Characterization of sex-independent SRBSDV-responsive lncRNAs in *Sogatella furcifera*

**Figure 5:**
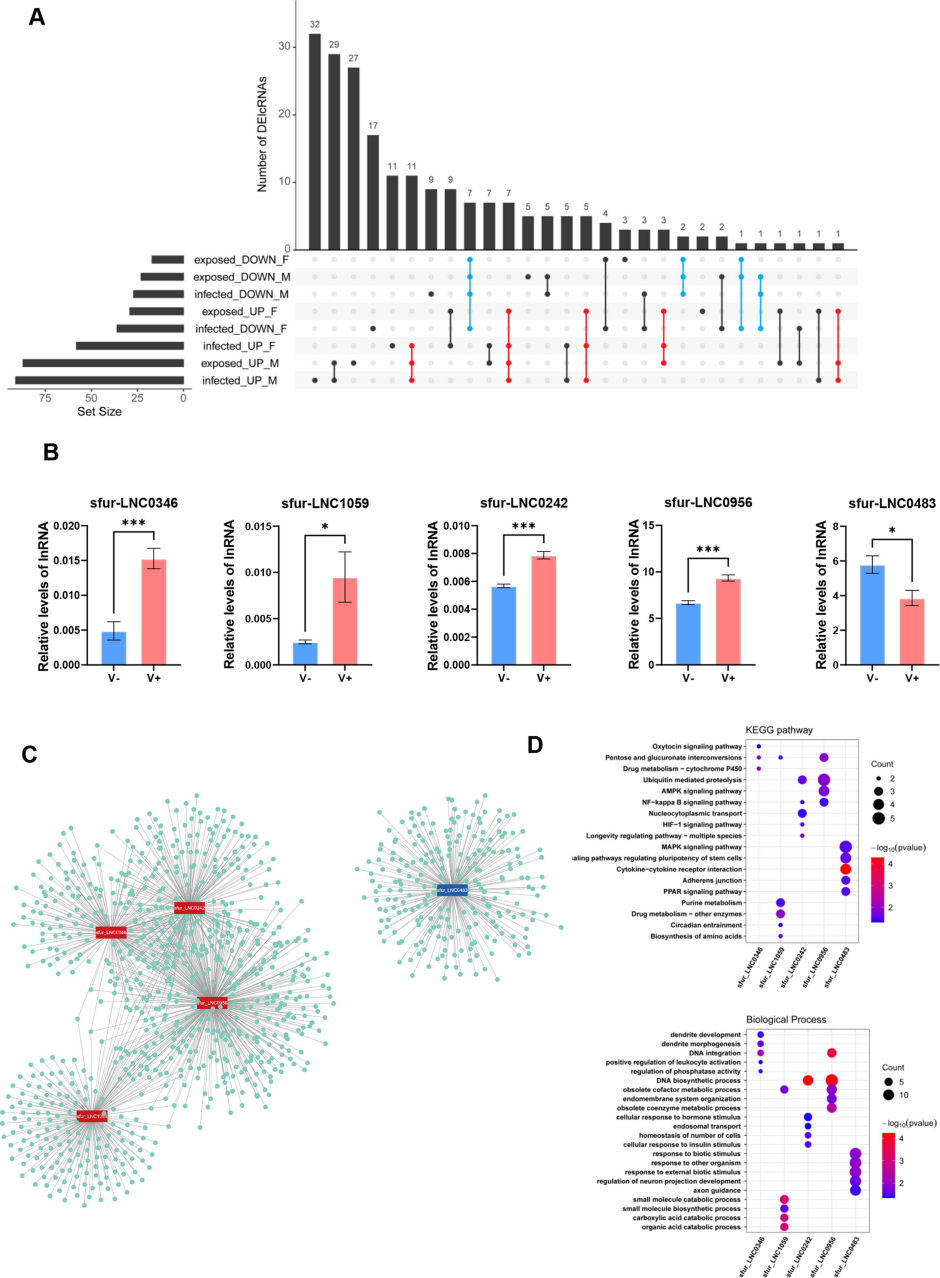
Experimental validation and functional prediction of DElncRNAs in WBPH upon the SRBSDV infection. **(A)** The robust, sex-independent DE-lncRNAs in *S. furcifera* upon virus infection. **(B)** Quantitative RT-PCR analyses of the expression of virus-responsive lncRNAs. The levels were normalized using actin transcripts as an internal control. The mean RT-qPCR assay results ± the standard deviations of three biological replicates are shown (*p < 0.05, ***p < 0.001, *t*-test). **(C)** The candidate lncRNAs and nearby co-expressed mRNA networks for functional prediction. **(D)** The KEGG and GO enrichment analysis of mRNAs that were highly correlated with the candidate virus-responsive lncRNAs.

Given that many lncRNA-mediated antiviral responses in *S. furcifera* appear to be sexually dimorphic, we aimed to identify robust lncRNAs that are differentially expressed in a sex-independent manner. To this end, we focused on lncRNAs that were consistently upregulated or downregulated across both sexes and all SRBSDV treatment conditions. This analysis identified 27 lncRNAs that were consistently upregulated and 11 that were consistently downregulated in response to viral infection (Figure 5A; Supplementary Table S8). For validation of sex-independent SRBSDV-responsive lncRNAs in *S. furcifera*, we selected 30 DElncRNAs (20 upregulated and 10 downregulated) for experimental verification by qRT-PCR using gene-specific primers (Supplementary Figure 3A; Supplementary Table S9). Among them, five lncRNAs exhibited reproducible and statistically significant differential expression: sfur_LNC0242, sfur_LNC1059, sfur_LNC0956, and sfur_LNC0346 were upregulated, while sfur_LNC0483 was downregulated following SRBSDV infection (Figure 5B). All five lncRNAs are classified as intergenic, located at least 1 kb away from the nearest protein-coding gene, with transcript lengths of 746 bp (sfur_LNC0242), 234 bp (sfur_LNC1059), 1,199 bp (sfur_LNC0346), 1,790 bp (sfur_LNC0956), and 2,275 bp (sfur_LNC0483) (Supplementary Figure 3B).

To explore their potential functional roles, we predicted putative *cis-and trans*-regulated mRNA targets of these five virus-responsive lncRNAs (Figure 5C). The four upregulated lncRNAs demonstrated strong co-expression with a broader set of target genes, suggesting they may participate in coordinated regulatory programs. In contrast, the downregulated lncRNA, *sfur_LNC0483*, exhibited fewer co-expressed targets, indicating a more independent regulatory role—consistent with its distinct expression dynamics and functional enrichment profile. Gene Ontology (GO) and KEGG pathway enrichment analyses of the predicted mRNA targets revealed significant enrichment in pathways related to signal transduction, immune response, metabolic regulation, and cellular differentiation (Figures 5D,E). Specifically, *sfur_LNC0242* and *sfur_LNC0956* were associated with mRNAs enriched in NF-κB signaling and ubiquitin-mediated proteolysis, implicating them in the regulation of innate immunity and inflammatory responses. In contrast, *sfur_LNC0483* was linked to the AMPK and PPAR signaling pathways, which are known to govern metabolic adaptation, suggesting a potential role in modulating host energy balance and stress response during viral infection.

## 4 Discussion

This study represents a significant advancement in the understanding of lncRNAs in *S. furcifera*, a major pest of rice crops. By applying two PacBio SMRT sequencing data, we achieved a comprehensive characterization of full-length lncRNAs, overcoming the limitations of previous short-read annotations, such as truncated transcripts and inaccurate splice junctions. The identified full-length lncRNAs exhibited hallmark features, including low expression and tissue specificity, consistent with other species (Gloss and Dinger, 2016; Zhang et al., 2024; Mouhou et al., 2025). Using this refined reference, we examined lncRNA expression dynamics across developmental stages and identified stage-specific, functionally relevant transcripts. Additionally, we uncovered distinct sex-biased and SRBSDV-responsive transcriptional modules, along with a set of robust, sex-independent virus-responsive lncRNAs. Collectively, our findings provide a valuable resource for lncRNA research in non-model insects and underscore the regulatory importance of lncRNAs in development, tissue specialization, and antiviral defense.

Previous studies on *S. furcifera* and related planthopper species have largely depended on short-read sequencing technologies, which are often limited in their ability to resolve full-length transcript architectures and detect novel isoforms. For example, Chang et al. (2020) identified only 1,861 lncRNAs in the white-backed planthopper (WBPH) using short-read RNA-seq—a substantially lower number compared to the 1,211 high-confidence, full-length lncRNA transcripts characterized in the present study. This striking difference underscores the superior capability of long-read sequencing in achieving comprehensive transcriptome annotation. By employing PacBio long-read sequencing, our study not only confirmed known lncRNAs but also uncovered previously undetected isoforms and splice variants, significantly expanding the repertoire of lncRNAs in planthoppers. Notably, the identified lncRNAs exhibit conserved features consistent with those reported in other species, lower GC content, shorter transcript lengths, and fewer exons compared to mRNAs, further validating their biological relevance (Chen J. et al., 2016; Zhang M. et al., 2023). Notably, alternative splicing (AS) events were rare in lncRNAs (only 21 detected), contrasting sharply with the extensive AS observed in mRNAs. This aligns with findings in *Drosophila* and *Bombyx mori*, where lncRNAs generally exhibit simpler splicing patterns (Chen B. et al., 2016; Wu et al., 2016; Zhou et al., 2016; Schor et al., 2018; Li et al., 2019). The predominance of intergenic and antisense lncRNAs suggests diverse regulatory mechanisms, including transcriptional interference and chromatin remodeling (Mattick, 2011).

Our analysis uncovered developmental stage-specific expression patterns of these 1,211 lncRNAs, implicating them in key developmental processes. We found 412 lncRNAs expressed during early embryogenesis, with expression levels increasing progressively from 0 to 72 h post-oviposition in both sexes. This temporal expansion in lncRNA diversity suggests their potential involvement in the regulation of stage-specific developmental programs. Notably, hierarchical clustering revealed sexually dimorphic expression patterns, with male and female embryos exhibiting distinct lncRNA profiles by 24 h. This divergence may reflect early sex-determination mechanisms or differential developmental trajectories in reproductive tissue formation. A subset of lncRNAs displayed a conserved expression pattern, peaking at 0 h and sharply declining thereafter. Conversely, the other subset of lncRNAs exhibited female-specific upregulation at later stages (24–72 h), coinciding with yolk deposition and oocyte maturation, implying a role in female reproductive development. Beyond embryogenesis, we identified 380 lncRNAs dynamically expressed during molting (pre-ecdysis, ecdysis, and post-ecdysis). Pathway analysis revealed that Tight junction, Cell cycle, and Wnt pathways were highly active prior ecdysis, which aligns with their known roles in epithelial remodeling and cuticle sclerotization (Truman, 2005; Moussian, 2010). Additionally, autophagy signaling were enriched in AE stages, likely facilitating tissue degradation and remodeling (Colombani et al., 2005; Antonova et al., 2012; Tettamanti et al., 2019). These observations suggest that lncRNAs may orchestrate the precise timing of molting-related gene networks, ensuring successful transition between nymphal instars. Collectively, the dynamic and sex-specific expression of lncRNAs in WBPH highlights their potential role in shaping developmental plasticity and reproductive strategies in hemipteran insects.

The virus-responsive lncRNAs in *S. furcifera* also exhibit conserved and sexually dimorphic expression patterns. Notably, 50% of differentially expressed lncRNAs were shared between virus-exposed (RT-PCR-negative) and infected groups, indicating that physiological cues from infected plants alone can trigger antiviral priming. Similar “non-infected” responses have been documented in aphids exposed to barley yellow dwarf virus-infected plants (Mao and Zeng, 2014; Chang et al., 2021), pointing to a conserved insect strategy to preempt viral invasion. Furthermore, a striking finding was the sexually dimorphic expression of lncRNAs upon viral infection. The pronounced sex-specific differences in lncRNA expression suggest divergent antiviral strategies between sexes (Figure 3). Similar sex-dimorphic immune responses have been reported in *Aedes aegypti* infected with dengue virus, where females exhibit stronger immune activation due to reproductive trade-offs (Reitmayer et al., 2021). This divergence suggests that lncRNAs may mediate sex-specific adaptations to infection, with implications for the design of sex-biased pest control strategies. For instance, viruses may exploit sex-specific behaviors—such as female phloem feeding for egg development—to optimize transmission. Additionally, plant defense mechanisms (e.g., phytohormone responses) might interact differently with male and female vectors, indirectly influencing viral transmission dynamics. The *k*-means clustering analysis revealed the suppression of Cluster 3/4 lncRNAs and their associated pathways (PI3K signaling, phagosome regulation, mitochondrial membrane, etc.) in infected individuals implies viral subversion of host membrane trafficking systems. This aligns with studies showing that plant reoviruses like SRBSDV hijack endosomal pathways for replication (Zhang et al., 2021; Zhang L. et al., 2023). Collectively, these findings underscore the potential for sex-specific lncRNAs to serve as targets for disrupting vector competence. For instance, RNAi-based silencing of female-biased lncRNAs linked to immune evasion could impair viral persistence, while targeting male-biased lncRNAs might reduce mating success or fertility. Additionally, the conserved, sex-independent lncRNAs (e.g., *sfur_LNC0242* and *sfur_LNC0956*) associated with NF-κB and ubiquitin pathways offer broad-spectrum targets for pest control strategies, given their consistent dysregulation across conditions.

This study establishes a foundation for functional lncRNA research in the white-backed planthopper (WBPH), underscoring their pivotal roles in regulating development and antiviral defense. These findings provide a springboard for future investigations into lncRNA-mediated host-pathogen interactions in agricultural pests. The conservation of lncRNA features across insects—such as low expression levels and tissue specificity—suggests evolutionary constraints on their regulatory functions. Comparative analyses with other planthoppers (e.g., *Nilaparvata lugens*) could reveal conserved lncRNAs involved in shared pathways, whereas divergent lncRNAs may underlie host-specific adaptations. Additionally, exploring the potential application of lncRNAs in RNAi-based pest control warrants further investigation. Targeting functionally relevant lncRNAs—such as female-biased lncRNAs to disrupt reproduction or immune-related lncRNAs—could offer novel strategies for pest management.

## Data Availability

The datasets presented in this study can be found in online repositories. The names of the repository/repositories and accession number(s) can be found in the article/Supplementary material.
