# Supplementary material for: Characterization of full-length long noncoding RNAs and identification of virus-responsive lncRNAs in Sogatella furcifera
Source: Front Microbiol. 2025 Jul 2;16:1643735. doi: 10.3389/fmicb.2025.1643735 (PMC12263661; doi:10.3389/fmicb.2025.1643735)
Supplement: Supplementary file 1 [file Image_1.PDF]

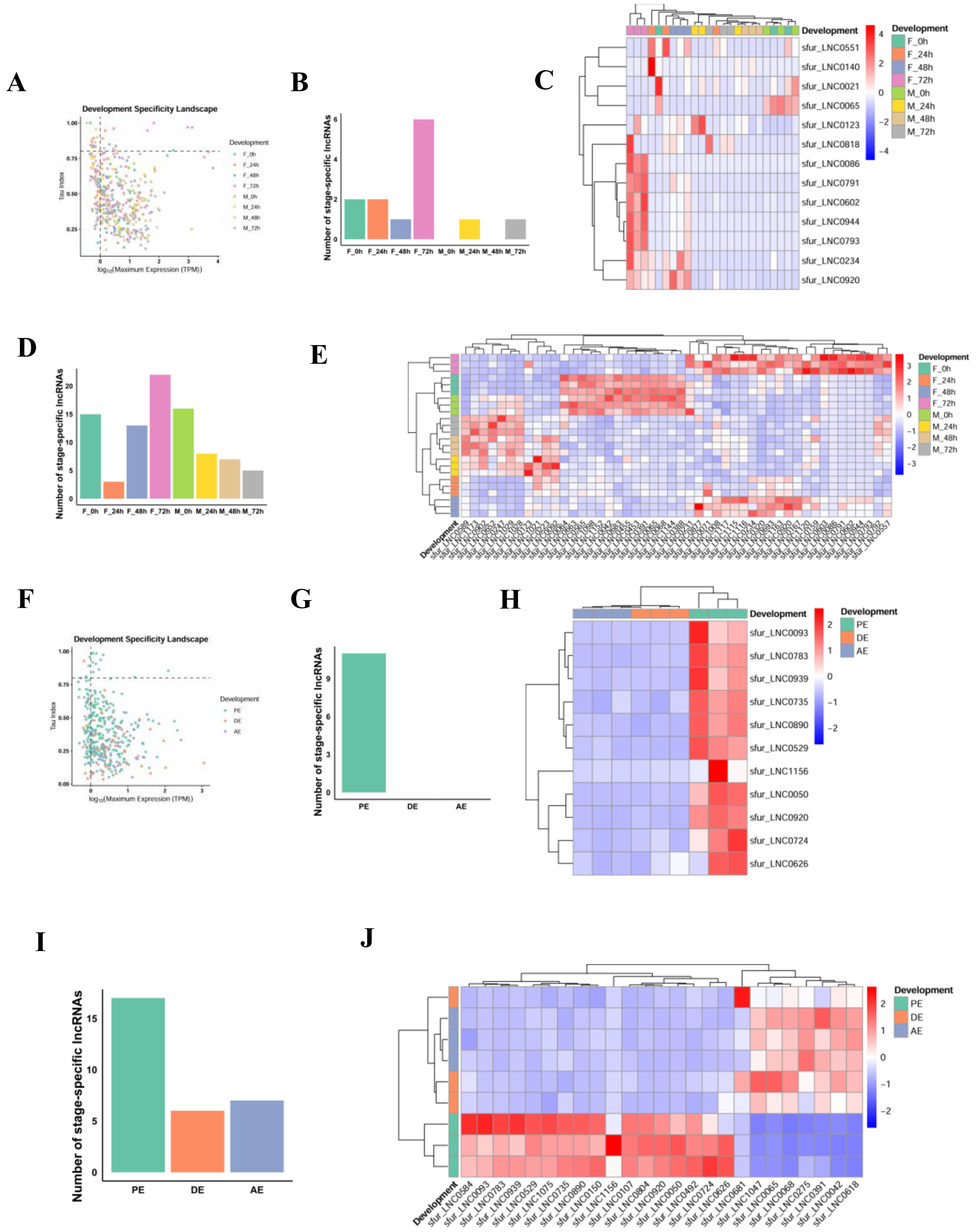


**Supplementary Figure 1** Tau values and differential expression profiles of lncRNAs in *Sogatella furcifera* across various developmental stages. A: Developmental stage-specific Tau value distribution of *S. furcifera*, lncRNAs with Tau > 0.8 and $\log_{10}$(TPM) > 0 are considered as developmental stages-specific gene candidates. B: Distribution of the number of developmental stages-specific lncRNAs screened by Tau. C: Heatmap of the expression profiles of developmental stages-specific lncRNAs screened by Tau. D: Distribution of the number of specific lncRNAs across various developmental stages screened by DEseq2 differential expression analysis. E: Heatmap of expression profiles of specific lncRNAs across various developmental stages screened by DEseq2 differential expression analysis. F: Ecdysis stage-specific Tau value distribution of *S. furcifera*; lncRNAs with Tau > 0.8 and $\log_{10}$(TPM) > 0 are considered as Ecdysis stage-specific gene candidates. G: Distribution of the number of tissue-specific lncRNAs screened by Tau. H: Heatmap of the expression profiles of Ecdysis stage-specific lncRNAs screened by Tau. I: Distribution of the number of specific lncRNAs across various ecdysis stages screened by DEseq2 differential expression analysis. J: Heatmap of expression profiles of specific lncRNAs across ecdysis stages screened by DEseq2 differential expression analysis.

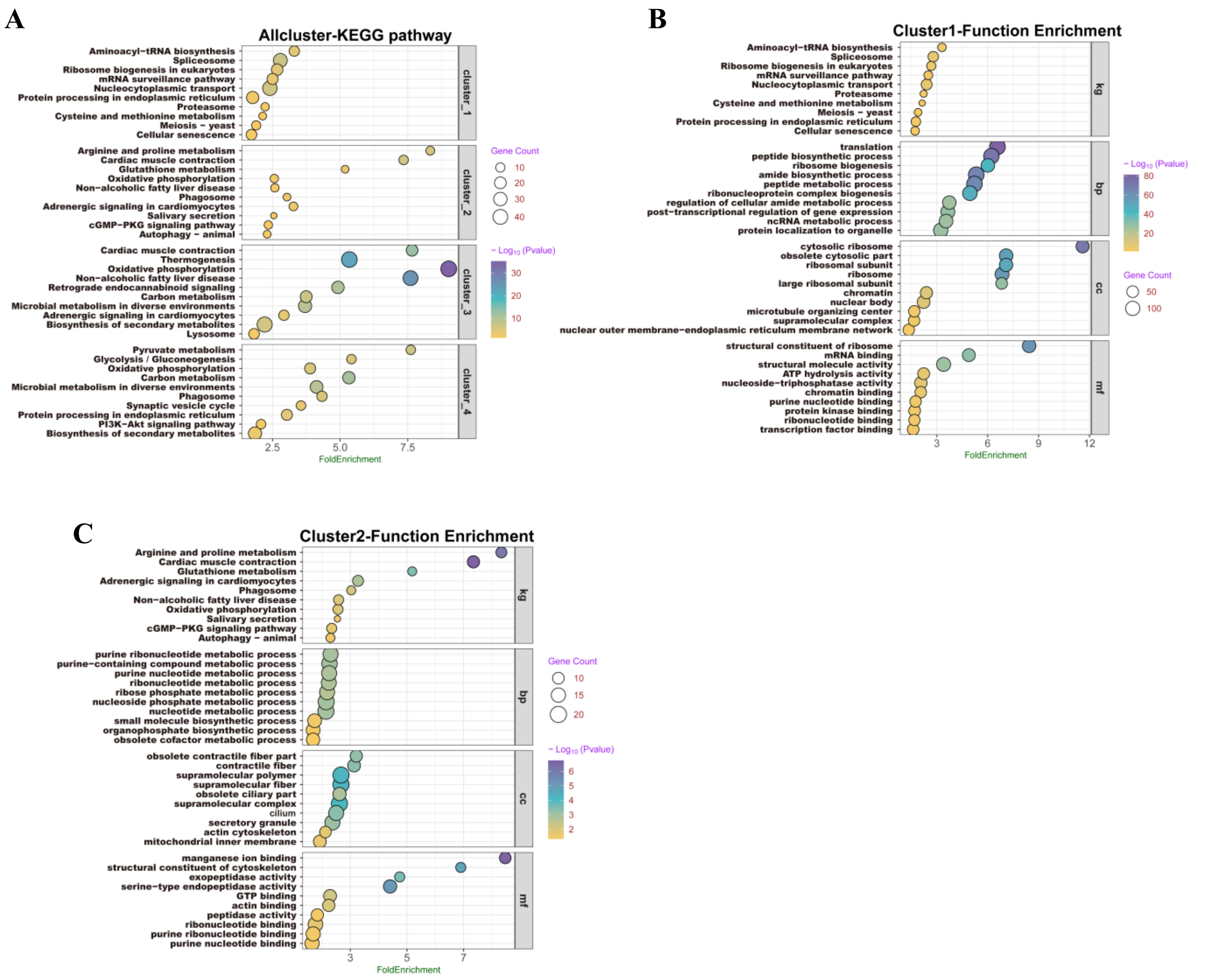


**Supplementary Figure 2** The enrichment analysis of the lncRNAs in four clusters through the k-means analysis. A: The KEGG pathway analysis of the lncRNAs in all four clusters in k-means analysis. B: Cluster1 is significantly enriched in translation-related processes, ribosome biogenesis, and mRNA regulatory pathways, supported by high gene counts (50–100) and diverse molecular functions, suggesting a central role in protein synthesis and post-transcriptional regulation. C: Cluster2 is significantly enriched in cardiac muscle functions, purine metabolism, and cytoskeletal structure, indicating active roles in cellular energy and nucleotide synthesis.

A

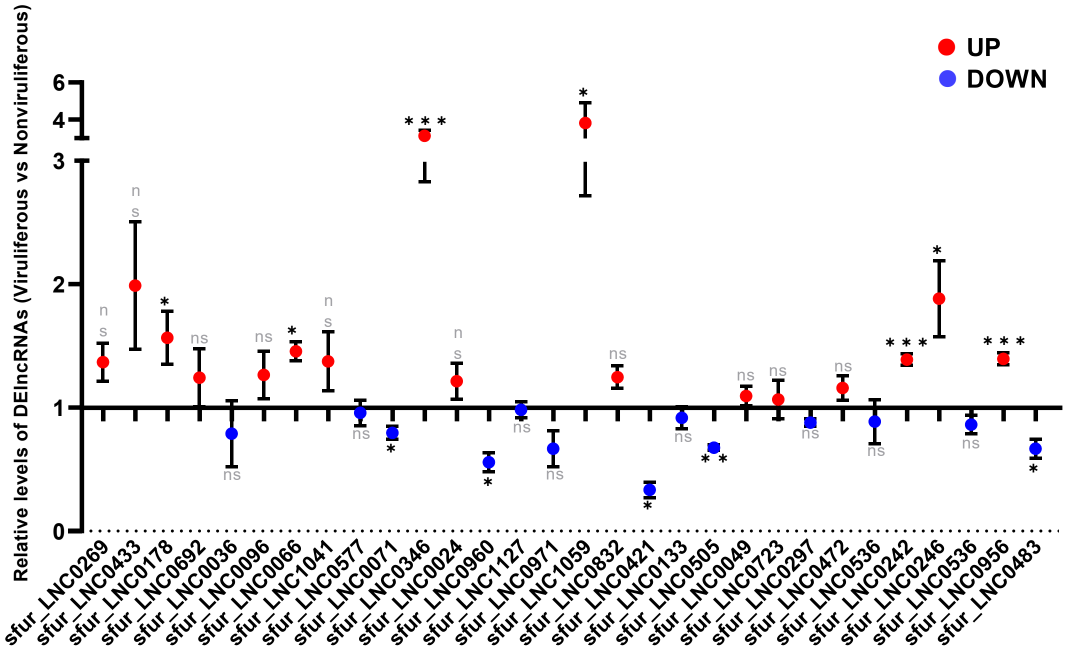


B

| DE-lncRNAs | Types | Lengh |
|---|---|---|
| sfur_LNC1059 | Intergenic | 234 |
| sfur_LNC0346 | Intergenic | 1199 |
| sfur_LNC0956 | Intergenic | 1790 |
| sfur_LNC0242 | Intergenic | 746 |
| sfur_LNC0483 | Intergenic | 2275 |

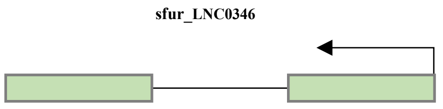


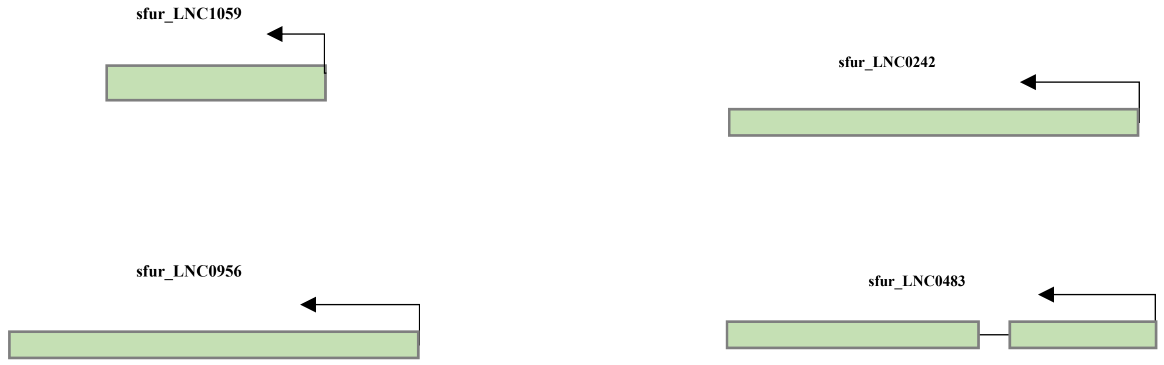


**Supplementary Figure 3** The differentially expressed lncRNA candidates (DElncRNAs) in WBPH upon the SRBSDV infection. A: For validation of SRBSDV-responsive lncRNAs in *S. furcifera*, we selected 30 DElncRNAs for experimental verification by qRT-PCR. B: The genomic structures and positions contexts of candidate virus responsive lncRNAs.
